# Knowledge, Attitude, and Associated Factors towards Physical Assessment among Nurses Working in Intensive Care Units: A Multicenter Cross-Sectional Study

**DOI:** 10.1155/2020/9145105

**Published:** 2020-08-10

**Authors:** Bikis Liyew, Ambaye Dejen Tilahun, Tilahun Kassew

**Affiliations:** ^1^School of Nursing, Department of Emergency and Critical Care Nursing, College of Medicine and Health Sciences, University of Gondar, Gondar, Ethiopia; ^2^School of Medicine, Department of Psychiatry, College of Medicine and Health Sciences, University of Gondar, Gondar, Ethiopia

## Abstract

**Introduction:**

Nurses working in the intensive care unit play an essential role in detecting patients at risk of deterioration through ongoing assessment and action in response to changing health status.

**Objectives:**

To assess knowledge, attitude, and associated factors towards physical assessment on critically ill patients among nurses working in the intensive care unit at Amhara regional state referral hospitals, Northwest Ethiopia, 2019. The research hypothesis: there is poor physical assessment knowledge, poor physical assessment attitude, and there are factors that are likely to affect nurses' knowledge and attitude towards physical assessment providing this care to critically ill patients at Amhara regional state referral hospitals, Northwest Ethiopia, 2019.

**Methods:**

Institution-based cross-sectional study was conducted among 299 nurses from March to September 2019. A convenience sampling method was used. Data were entered by using Epi Info 7.2.2 and analyzed by using STATA 14. The result was computed by descriptive statistics and to explore predictors of knowledge, and attitude linear regression analysis models were fitted, and the adjusted unstandardized beta (*β*) coefficient at 95% CI was used. A *p*-value <0.05 was considered significant. Result and conclusion: the knowledge mean scores were 9.93 ± 2.99 [95% CI (9.59, 10.31)]. The proportion of nurse's knowledge who score above the mean was 167 (55.9%) [95% CI (50.2, 61.5)] and below the mean 132 (44.1%) [95% CI (38.5, 49.8)]. Attitude means scores were 36.85 ± 6.21 [(36.16, 37.51)]. The proportions of nurse's attitudes who score above the mean were 158 (52.8%) [95% CI (47.5, 58.5)] and below the mean 141 (47.2) [95% CI (41.5, 52.5)]. Regarding predictor variables, being male [*β* = 0.84, 95% CI (0.16, 1.52)] and taken training [*β* = 1.85, 95% CI (1.14, 2.56)] were factors positively associated with knowledge, whereas has taken training [*β* = 4.13, 95% CI (2.82, 5.44)], total years of experience [*β* = 0.59, 95% CI (0.25, 0.93)], and knowledge [*β* = 0.92, 95% CI (0.0.72, 1.12)] were factors positively associated with attitude towards physical assessment.

**Conclusion:**

Based on the result of this study, the knowledge and attitude towards physical assessment regarding critically ill patients among nurses working in intensive care units were good. Hence, training, educational support services, and awareness are recommended to encourage nurse's knowledge and attitude towards physical assessment.

## 1. Introduction

Nurses working in intensive care units play an essential role in detecting patients at risk of deterioration through ongoing assessment and action in response to changing health status. Yet, evidence suggests that clinical deterioration frequently goes unnoticed in hospitalized patients. While much attention has been paid to early warning and rapid response systems [1, 2]. Physical assessment is an organized systemic process of collecting objective and subjective data based upon a health history and head-to-toe or general body systems examination [3]. Evidence suggests that lack of knowledge was the contributing factor for suboptimal care for acutely ill patients [4–7]. Some nurses do not consider physical assessment as part of their work. Today, there are multiple changes in the health care system; physical assessment is an important part of the definition of the physician's job, but it also becomes an integral part of the definite job of nurses [8]. The health assessment constitutes one of the key components in nursing skills and plays a decisive role in identifying the problems in different patient systems and the development of nursing care programs [9].

Clinical decision-making ability of nurses can affect the caretaking quality more than any other factor, and this ability depends on different skills, including health assessment. The most successful nurses are those who have high assessment skills, who have high training skills, and who are highly enthusiastic to use technology [10]. Nurses should act independently to achieve nursing goals and enjoy self-confidence in using health assessment skills [11]. Nurses are commonly and continuously in complex medical conditions that require advanced examination skills from the needs [12]. Intensive nursing care requires an assessment of patients, designs, and presentation of designs based on the information obtained from physical examination, interviews, and discussions on the patient's history [13]. ICU care demands a high level of expertise in many aspects because critically ill patients are patients who are at high risk for actual or potential life-threatening health problems. All care given by nurses depends on the finding from the physical assessment. Besides, inexperienced ICU nursing staff will hurt the quality of care of critically ill patients [11].

Nurses in the USA, and more recently Canada and Australia, readily incorporate physical assessment skills into their nursing practice as a component of health assessment [14]. Assessing the critically ill patient and family begins from the moment the nurse is made aware of the pending admission of the patient and continues until transiting to the next phase of care. Crucial to developing competence in assessing critically ill patients and their families is a consistent and systematic approach to assessment without this approach, it would be easy to miss subtle or details that may identify actual or potential problems and also indicate a patient's changing status [15, 16]. The findings of a nursing assessment do sometimes contribute to the identification of medical diagnosis; the unique focus of a nursing assessment is on the patient's responses to actual or potential problems [15].

The intensive care unit is the heart and main component of a clinical care setting. Due to the urgent conditions of patients who are hospitalized in ICUs, critical care nurses need to have great professional knowledge and experience, high-quality critical care, high technical equipment, great clinical competence staff, and great abilities in working with group decision-making to facilitate critically ill patients' recovery [17]. Nurses who base their practice on the scientific evidence and documents make wiser decisions, provide care services with higher quality, shorten patients' hospital stay, lower their healthcare costs, and improve care and organizational effectiveness. Different works of literature found that nurses had positive attitudes towards evidence-based practice [17–19].

There is knowledge about what intensive care patients experience as strengthening and empowering when being cared for in the ICUs, but there is still a lack of knowledge about how patients rate the importance of physical assessment practices, i.e., what is experienced to be of the greatest importance and what is not that important. A combination of patient-rated importance and actual experiences could serve as a basis for reflections and tailored improvement activities [20].

Knowledgeable and skillful critical care staff is a key component of high-quality critical care delivery. Critical care staff, particularly nurses, need to manage unpredictable critical situations and thus, they need to have adequate professional knowledge and skills. Another study also reported that more experienced nurses less frequently used theoretical academic knowledge. In other words, the use of theoretical knowledge was negatively correlated with the work experience [21, 22].

Some literature finding showed that more experienced nurses were more likely knowledgeable about physical assessment skills. Physical assessment skills were cited as more difficult to carry out by respondents with less experience in nursing. The use of evidence-based nursing practice is not only a duty but also a professional responsibility and practice. Evidence-based practice helps nurses have the best clinical practice and, thereby, improves the quality and effectiveness of nursing care services [15, 23, 24]. However, nurses who have a higher level of education have been shown to provide better nursing care, with higher levels of safety for their patients. Such competent performance requires the integration of nursing knowledge accounting for better decision-making and improved clinical reasoning and performance [25].

In another study, relatively low skill was utilized by second-year bachelor of nursing students following a physical assessment course. Students generally used inspection and where body systems were assessed, only skin assessment was frequently conducted. Pre- and postmeasures of attitudes towards health assessment showed a significant positive change, which also correlated positively with skill usage [26].

Clinical frontline nurses play an essential role in detecting changes in patients' health status through an ongoing health assessment and, timely, appropriate action in response to changes, or deterioration, in health status [1]. Despite the centrality of health assessment in nursing education, previous research suggests that only 11–29% of the physical assessment skills taught in nursing programs are regularly used by RNs in practice [13]. Questions were raised about the need for nursing students to learn such a large range of physical assessment skills to practice nursing skills, which were derived from a medical model and whereby only a small set of these skills were used in practice [27].

Despite this, Ethiopia's populations still face a high rate of morbidity and mortality. Nurses are the key caregivers in hospitals; they can significantly influence the quality of care and, ultimately, treatment and patient outcomes through physical assessment. Despite physical assessment, many nurses have stated that this is not part of their job and perceived negatively [15].

Critically ill patients need an advanced modern approach to care to depend on finding the physical assessments of individuals. Knowledgeable skill with a positive attitude is essential to guide correct nursing action plan based physical examination finding. Therefore, to assess knowledge, attitude, and factors among nurses towards physical assessment on critically ill patients are primarily used to improve the quality of care in ICU patients.

## 2. The Research Hypotheses

1. There is poor physical assessment knowledge among nurses working in the intensive care unit providing this care to critically ill patients at Amhara regional state referral hospitals, Northwest Ethiopia, 2019.
2. There is poor physical assessment attitude among nurses working in the intensive care unit providing this care to critically ill patients at Amhara regional state referral hospitals, Northwest Ethiopia, 2019
3. Some factors are likely to affect nurses' knowledge and attitude towards physical assessment providing this care to critically ill patients at Amhara regional state referral hospitals, Northwest Ethiopia, 2019.

## 3. Methods

### 3.1. Study Setting and Populations

The institutional-based cross-sectional study design was maintained from May to September 2019 at Amhara regional state referral hospitals. The population of this study consisted of 299 nurses employed at the AICU, NICU, and PICU of Amhara regional state referral hospitals. ARS has 42 hospitals, among these there are 5 referral hospitals. The five referral hospitals are Felege Hiwot, Dessie, Gondar, Debre Berhan, and Debre Markos. All nurses' works in ICU at Amhara regional state referral hospitals were included in this study. The inclusion criteria included nurses who were working in ICU for at least six months and above. The exclusion criteria for this study were the nursing personnel not involved in the direct management of the patients (e.g., nursing managers and tutorial staffs) were excluded.

### 3.2. Data Collection Procedures and Instruments

The study tools were developed by three Ethiopian emergency medicine and critical care nursing educators and three nurses currently practicing in the clinical area and from previous gray works of literature (unpublished) [28]. The questionnaire was designed as per the standard module and practices of tool and questionnaire development [29]. The researcher examined the questionnaire for content and face validity, clarity, and discrimination of items. These tools have three parts such as sociodemographic characteristics of study respondents, knowledge, and attitude of nurses toward physical assessment of critically ill patients using well-structured questionnaire and self-administered response methods.

  Section one: Sociodemographic characteristics of study respondents were involved.
  Section two: The physical assessment knowledge questionnaires were used to measure the knowledge of ICU nurses toward physical assessment, which consisted of 15 multiple-choice questions, and taken from physical assessment questionnaires. After tremendous searching and discussion of a nurse's scope of practice and reviewing different literature. The correct answer of each item was scored as 1 and incorrect answer scored as 0. The possible range of total score of knowledge on physical assessment was 0–15. Nurses with the total score closer to 0 indicate very poor knowledge and those with a total score closer to 15 indicate the best possible knowledge
  Section three: The attitude was measured through the original questionnaire with modification from previous literature which consists of 10 items. Five of the items were worded positively (1 = strongly disagree to 5 = strongly agree), and five were phrased as negatively. The negative Likert scale questions were reversely coded (5 = strongly disagree to 1 = strongly agree). Thus, the possible score range was 10 to 50. Nurses with the total score closer to 10 indicate very poor attitude and those with a total score closer to 50 indicate the best possible favorable attitude about physical assessment skill.

Data were collected by using a structured self-administered questionnaire. The data were collected by four Ethiopian research assistants and collectors with three BSc nursing qualifications. During the actual data collection process, the supervisor has cross-checked the completeness and well fill of the data consistently. The data were cleaned from inconsistencies and missing values, and the amendment was considered as needed before data analysis. A pretest was performed to ensure the reliability and validation of study tools. Content and face validity was evaluated by a panel of six nurses with expertise in the area, including nurse managers, educators and researchers from the target population, and nursing academics responsible for teaching undergraduate and postgraduate health assessment, after which one final modification was made.

### 3.3. Data Processing and Analysis

The collected data were entered into Epi Info version 7.2 and analyzed by STATA version 14. Descriptive statistics were used to summarize the sample characteristics. Assumption tests and simple linear regression analysis were performed to determine the correlation of each independent variables with knowledge and attitude. Those variables with *p*-value <0.2 during the analysis were selected for multiple linear regression analysis, and model fitness tests were (*R*^2^) also performed. The result was expressed as an adjusted “*ß*” coefficient. About 95% confidence level was employed to determine the factors associated with knowledge and attitude regarding physical assessment. A *p*-value of <0.05 was considered statistically significant.

## 4. Results

### 4.1. Sociodemographic Characteristics of Study Participants

Among 299 nurses recruited in the study, more than half of the study participants were female 162 (54.2%). The mean age of the participants was 31.9 (±3.808) years. Two hundred thirty-six (78.9%) participants were married. The majority of the study participants 236 (88%) were orthodox Christian. From a total of 299 study participants, the average monthly income was 5748.64 (±1698.42) Ethiopian birr. The mean total years of work experience were 5.7 ± 2.54, and years of experience working in ICU were 1.83 ± 0.798 (Table 1).

### 4.2. Knowledge of ICU Nurses towards Physical Assessment

Out of 299 nurses working in intensive care units, the knowledge mean scores were 9.93 ± 2.99 (9.59, 10.31) with a 95% confidence interval. The proportion of nurses knowledge who score above the mean was 167 (55.9%) with 95% CI (50.2, 61.5) and below the mean 132 (44.1%) with 95% CI (38.5, 49.8). The minimum and maximum knowledge scores were 3 and 15, respectively.

One hundred eighty-one (60.5%) of the ICU nurses' correct answers react to the relation of RR assessments. The presence of bruits in the carotid artery may suggest turbulent blood flow or stenosis was answered only by 122 (40.8%) of ICU nurses. 122 (40.8%) of study respondents identify the types of normal and abnormal breathing sounds. However, about 177 (59.2%) of ICU nurses could not identify the location of those normal breathing sounds find during chest auscultation. Additional heart sound S3 can be heard during ventricles are resistant to fill correctly was answered only by 172 (57.5%) of ICU nurses. From total ICU nurses, only about 158 (52.8%) and 66 (22.1%) had known types of pitting edema and stage of pressure ulcer respectively. More than half, 186 (62.2%), of the study respondents had known common signs and symptoms of respiratory dysfunction during respiratory complaints of critically ill patients (Table 2).

### 4.3. The Attitude of ICU Nurses towards Physical Assessment

From 299 nurses working in intensive care units, the attitude mean scores were 36.85 ± 6.21 (36.16, 37.51) with a 95% confidence interval. The proportions of nurses' attitude who score above the mean were 158 (52.8%) with 95% CI (47.5, 58.5) and below the mean 141 (47.2) with 95% CI (41.5, 52.5). The minimum and maximum scores of attitude were 21 and 49, respectively.

About 120 (40.1%) ICU nurses strongly agree with head-to-toe physical examination for critically ill patients is very important. 43 (14.4%) study participants strongly agree with physical assessment for critically ill patients on mechanical ventilation is very difficult. Around 50 (16.7%) study respondents strongly agree on physical assessment always performed by a physician as a trained experience. Twenty-six (8.7%) ICU nurses strongly disagree with ideas on routine physical assessments for critically ill patients as are the responsibility of nurses. About 92 (30.8%) of respondents strongly agree with in ICU daily physical assessment to result in a new diagnosis, this may change the diagnosis and treatments of individuals on critically ill patients. Around 28 (9.4%) ICU nurses believe that physical assessment is not a nursing job. A vast majority of 120 (40.1%) of the nurses in this study agreed that head-to-toe physical examination for critically ill patients is important (Table 3).

### 4.4. Factors Associated with Knowledge of Nurses Working in ICU towards Physical Assessment

In simple linear regression analysis, it was indicated that taken training, age, total year of experiences, and year of experience in ICU were factors positively associated with the total knowledge score of nurses towards physical assessment at the *p*-value of 0.05. Multiple linear regressions showed that being male (*β* = 0.84, 95% CI (0.16, 1.52)) and had taken training (*β* = 1.85, 95% CI (1.14, 2.56)) were factors positively associated with knowledge towards physical assessment among nurses (Table 4).

### 4.5. Factors Associated with the Attitude of Nurses Working in ICU towards Physical Assessment

In simple linear regression analysis, it was indicated that taken training, total years of experience, and knowledge were factors positively associated with the total knowledge score of nurses towards physical assessment at a *p-*value of 0.05. In multiple linear regressions, it was shown that taken training (*β* = 4.13, 95% CI (2.82, 5.44)), total years of experience as a nurse (*β* = 0.59, 95% CI (0.25, 0.93)), and knowledge (*β* = 0.92, 95% CI (0.0.72, 1.12)) were factors positively associated with the attitude towards physical assessment among nurses (Table 5).

## 5. Discussion

This study was carried out to assess knowledge, attitude, and associated factors towards physical assessment on critically ill patients among nurses working in the intensive care unit at Amhara regional state referral hospitals. There is growing evidence of failure to recognize hospitalized patients at risk of clinical deterioration, in part due to inadequate physical assessment knowledge and attitude by nurses [30, 31]. There is limited literature related to knowledge and attitude towards physical assessment among nurses in acute care settings, and it is challenging to make a comparison without consistent measuring instruments with little studies that do exist [32, 33].

The findings showed that nurses working in ICU had better knowledge and favorable attitudes towards physical assessment in critically ill patients. Out of 299 nurses working in intensive care units, the knowledge mean scores were 9.93 ± 2.99 (9.59, 10.31) with 95% confidence interval. However as far as our literature searching effort, there is no study done before similar to our topic and the nature of outcome rating which is mean score of knowledge. Besides this, the proportion of nurses' knowledge who score above the mean was 167 (55.9%) with 95% CI (50.2, 61.5) and below the mean 132 (44.1%) with 95% CI (38.5, 49.8). This finding is higher than in previous studies [11, 20, 34]. The possible reason for the difference might be due to the approach to the summation of the outcome variables, the difference in measuring tools, sample size difference, the study design, sociocultural differences, data collection technique, and difference between participants. In the current study, the total scoring of the knowledge items was by giving 0 for incorrect and 1 for correct answers, which ranged from 0 to 15, whereas in Sweden and Australia qualitative research approach and literature review and a concept analysis-based method were used, respectively. The sample size was higher in this study (*n* = 299) compared to that of Australia (*n* = 208). The study participants in this study were nurses working in intensive care units, whereas in Australia they were graduating nurses. Therefore, this finding is supposed to increase the mean score of knowledge towards physical assessment. The current study was a large multicenter cross-sectional study, whereas in Australia it was single-center cross-sectional study design.

The attitude mean scores of nurses working in the intensive care unit were 36.85 ± 6.21 with 95% CI (36.16, 37.51). The proportions of nurses attitude who score above the mean were 158 (52.8%) with 95% CI (47.5, 58.5) and below the mean 141 (47.2%) with 95% CI (41.5, 52.5). This finding is higher than in previous studies [17–19]. The possible justification for this study might be due to sociocultural differences, data collection techniques, and differences between participants.

Regarding predictor variables of knowledge, as being male (*β* = 0.84, 95% CI (0.16, 1.52)) increased by a unit, knowledge of nurses towards physical assessment increased by 0.84 units as compared with female nurses. Females nursing students recorded higher barriers in physical assessment than males [34]. As had taken training (*β* = 1.85, 95% CI (1.14, 2.56)) increased by a unit, knowledge towards physical assessment increased by 1.85 units as compared with did not receive training about physical assessment. Training of nurses plays an important role in improving the quality of patient care. The need to promote the effectiveness of in-site and off-site training of nurses is an invaluable criterion. Training is necessary to update theoretical and practical knowledge in every aspect of nursing education [35, 36].

According to the present study findings as had taken training (*β* = 1.85, 95% CI (1.14, 2.56)) increased by a unit, knowledge towards physical assessment increased by 1.85 units. Moreover, the current study is supported by a study conducted in Victoria, Australia. Inadequately trained staff in health assessment may not be encouraged to conduct physical assessment skills. Untrained nursing staffs point out barriers than enablers to implement physical assessment in practice [37]. The possible reason might be training on specialty and educational curriculum internationalization. But the current study findings revealed that no significant association could be demonstrated between knowledge score and nurses' age, educational level, total years of experience as a nurse, years of experience in ICU, and marital status. This study is inconsistent with some literature findings showed that nurses with more experience were more knowledgeable about almost all physical assessment skills. The group with more clinical practice experience had more knowledge of physical assessment skills, used the skills more frequently, and had less difficulty in using them [11]. Besides, another study reported that more experienced nurses less frequently used theoretical academic knowledge on physical assessments [21, 22]. This is due to different influencing factors to affect patient assessments include nurses' perceived lack of knowledge, a lack of confidence in practice, and lack of experience. Knowledgeable and skillful critical care professionals are a key component of high-quality critical care delivery. Critical care nurses need to manage and prevent unexpected acute care conditions and thus, they need to have good health care knowledge and practice [21, 22].

Regarding predictor variables of attitude, had taken training (*β* = 4.13, 95% CI (2.82, 5.44)) increased by a unit: attitude towards physical assessment among ICU nurses was increased by 4.13 units as compared with those who had not taken the training. Nursing is not simply the ability to give quality care, rather, nursing is a holistic practice, including psychological, social, environmental, and spiritual aspects of an illness and its impact on patients and their relatives. Therefore, effective and organized training has been recognized as the key way to change nurses' effective communication with patients [38, 39].

The result of the current study also showed that total years of experience as a nurse (*β* = 0.59, 95% CI (0.25, 0.93)) increased by one unit: attitude towards physical assessment increased by 0.59 units. In the current study, by considering the other variables constant as knowledge (*β* = 0.92, 95% CI (0.0.72, 1.12)) increased by a unit, attitude towards physical assessment increased by 92%. One of the basics for quality nursing care in nursing education is to consist of the three domains of learning: knowledge, attitude, and practice. If the attitude of nursing professionals is not good, the quality of health care could be questionable. So, increasing nurses' cognitive domain will increase awareness and reduce lack of interest in clinical settings, indirectly improving the quality of care by systematic, advanced physical assessment techniques [40–42]. A better attitude towards clinical practice enhances effective clinical learning, whereas a negative attitude hinders the acquisition of necessary clinical practice. So, determining the discrepancy in clinical practice is noteworthy for enhancing the quality of nursing educations.

Our study result showed that educational status had not a significant association with attitude and knowledge of nurses towards physical assessment. This is inconsistent with another study, which elaborates that nurses who have a good level of qualification have been shown to provide better health care, with higher levels of safety for their patients. Such competent performance needs integration of nursing knowledge and practice for better clinical practice decision-making and improved clinical reasoning and performance [25]. Knowing enough about physical assessment in an acute care setting is mandatory for the professional development of health care providers that would enhance, promote, and encourage nurses to work independently and to increase the quality of health care delivery.

## 6. Conclusion and Recommendation

Based on the finding of this study, it was shown that nurses working in ICU had better knowledge and favorable attitudes towards physical assessment skills in critically ill patients. Regarding predictor variables, being male and had taken training were factors positively associated with knowledge towards physical assessment among nurses working in intensive care units, whereas had taken the training, total years of experience as a nurse, and knowledge were factors positively associated with the attitude towards physical assessment. Hence, training on physical assessment towards critically ill patients and educational support services and awareness are recommended to increase nurse's knowledge and attitude towards physical assessment.

## 7. Limitations of the Study

Since the study was based on self-reported data in estimating the knowledge and attitude of nurses towards physical assessment, it is a common threat to the validity of the self-report that can lead to information bias such as social desirability bias. Besides, a cross-sectional study by its nature cannot establish a definitive cause and effect relationship to identify the risk factors.

## Figures and Tables

**Table 1:** Sociodemographic characteristics of study participants at Amhara regional state referral hospitals, Northwest Ethiopia, 2019.

Variables	Categories	Frequency (N = 299)	Percentage (%)
Sex	Male	137	45.8
	Female	162	54.2

Marital status	Married	236	78.9
	Unmarried	63	21.1

Religion	Orthodox	263	88.0
	Muslim	21	7.0
	Protestant	15	5.0

Educational level	Diploma	8	2.7
	Degree	249	83.3
	Master	42	14.0

Work area currently employed	Adult ICU	190	63.5
	Pediatric ICU	22	7.4
	Neonatal ICU	87	29.1

Training	Yes	115	38.5
	No	184	61.5

ICU = intensive care unit.

**Table 2:** Study participant's knowledge towards physical assessment at Amhara regional state referral hospitals, Northwest Ethiopia, 2019.

Knowledge questionnaire items	Responses	
	Correct *n* (%)	Incorrect *n* (%)
RR should be assessed concerning:-	181 (60.5)	118 (39.5)
The presence of bruits in the carotid artery may suggest	122 (40.8)	177 (59.2)
Normal breathing sound includes	122 (40.8)	177 (59.2)
S3 heart sound can be heard when	172 (57.5)	127 (42.5)
Breast tissue does not change with aging	242 (80.9)	57 (19.1)
Pitting edema that disappears within a few seconds considered as	158 (52.8)	141 (47.2)
Stage 3 pressure ulcer injuries involve	66 (22.1)	233 (77.9)
Normal body temperature varies throughout the day.	248 (82.9)	51 (17.1)
Usually, blood pressure in the left and right arm differs by more than 15 mmHg	126 (42.1)	173 (57.9)
The most common sign and symptom of respiratory dysfunction is	186 (62.2)	113 (37.8)
The definition of orthopnea is needed to sit or stand to breathe normally	127 (42.5)	172 (57.5)
Does addition breathing sound like wheeze and stridor are common on further what data gathering needed for the above case (aortic aneurism)?	179 (179)	120 (40.1)
What additional information related to this recent development?	243 (81.3)	56 (56)
What complications will happen for the above case (aortic aneurism)?	21 (7.0)	278 (93.0)
Which one is mismatched about normal breathing sound with location?	87 (29.1)	212 (70.9)

RR = respiratory rate; mmHg = millimeter mercury.

**Table 3:** Attitude of study respondent's assessment at Amhara regional state referral hospitals, Northwest Ethiopia, 2019.

Attitude related questionnaire items	Response				
	Strongly disagree *N* (%)	Disagree *N* (%)	Neutral *N* (%)	Agree	Strongly agree *N* (%)
Head-to-toe PE for critically ill patients is important	44 (14.7)	11 (3.7)	38 (12.7)	86 (28.8)	120 (40.1)
Critical ill patients on mechanical ventilation PE is a difficulty	45 (15.1)	58 (19.4)	53 (17.7)	100 (33.4)	43 (14.4)
Physical assessment always performed by a physician	88 (29.4)	90 (30.1)	32 (10.7)	39 (13.0)	50 (16.7)
Routine PE for critically ill patients is the responsibility of nurses	26 (8.7)	75 (25.1)	14 (4.7)	127 (42.5)	57 (19.1)
In ICU daily PE to result in new d*x*, this may change d*x* and treatments	36 (12.0)	44 (14.7)	37 (12.4)	90 (30.1)	92 (30.8)
Physical assessment is not nursing jobs	108 (36.1)	98 (32.8)	33 (11.0)	32 (10.7)	28 (9.4)
Critically ill patients have less outcome, then daily PE is unnecessary	138 (46.2)	44 (14.7)	35 (11.7)	60 (20.1)	22 (7.4)
Physical assessment for critically ill patients to take a long time	110 (36.8)	65 (21.7)	63 (21.1)	25 (8.4)	36 (12.0)
In ICU, there is work overload, then PE is not more necessary	67 (22.4)	135 (45.2)	29 (9.7)	47 (15.7)	21 (7.0)
Always PE for critically ill patients is not more important	95 (31.8)	96 (32.1)	43 (14.4)	33 (11.0)	32 (10.7)

PE = physical examination; *D*_x_ = diagnosis; and ICU = intensive care units.

**Table 4:** Factors associated with knowledge towards physical assessment at Amhara regional state referral hospitals, Northwest Ethiopia, 2019 (*n* = 299).

Variables	Categories	Crude unstandardized *β* coefficient (95%CI)	Adjusted unstandardized *β* coefficient (95% CI)
Sex	Female	0	
	Male	0.55 (−0.13, 1.23)	0.84 (0.16, 1.52)^*∗∗*^
Marital status	Married	0	0
	Unmarried	−0.39 (−1.12, 0.34)	−0.21 (−0.94, 0.52)
Educational level	Diploma	0	
	Degree	−1.08 (−3.19, 1.03)	−0.57 (−2.67, 1.52)
	Master	−1.21 (−3.49, 1.06)	−0.89 (−3.10, 1.33)
Training	No	0	0
	Yes	1.89 (1.23, 2.56)^*∗*^	1.85 (1.14, 2.56)^*∗∗*^
Age in years		0.14 (0.06, 0.23)^*∗*^	0.05 (−0.08, 0.19)
Total years of experience		0.18 (0.05, 0.32)^*∗*^	0.02 (−0.17, 0.21)
Years of experience in ICU		0.54 (0.11, 0.96)^*∗*^	0.26 (−0.23, 0.75)

*Note*. ^*∗*^Significant at *p* < 0.05 (crude unstandardized *β* coefficient (95% CI)). ^*∗∗*^Significant at *p*-value <0.05 (adjusted unstandardized *β* coefficient (95% CI)).

**Table 5:** Factors associated with the attitude of nurses towards physical assessment at Amhara regional state referral hospitals, Northwest Ethiopia, 2019 (*n* = 299).

Variables	Categories	Crude unstandardized *β* coefficient (95% CI)	Adjusted unstandardized *β* coefficient (95% CI)
Sex	Female	0	0
	Male	−0.73 (−2.14, 0.69)	−0.88 (−2.09, 0.34)
Marital status	Married	0	0
	Unmarried	−0.99 (−2.51, 0.53)	−0.59 (−1.89, 0.70)
Educational level	Diploma	0	0
	Degree	−0.46 (−4.86, 3.95)	2.63 (−1.07, 6.33)
	Master	−0.11 (−4.84, 4.63)	3.89 (−0.04, 7.79)
Training	No	0	0
	Yes	5.37 (4.05, 6.69)^*∗*^	4.13 (2.82, 5.44)^*∗∗*^
Age in years		0.13 (−0.05, 0.38)	0.15 (−0.09, 0.38)
Total years of experience		0.03 (0.25, 0.31)^*∗*^	0.59 (0.25, 0.93)^*∗∗*^
Years of experience in ICU		0.65 (−0.23, 1.54)	0.15 (−0.71, 1.01)
Knowledge		1.07 (0.87, 1.27)^*∗*^	0.92 (0.72, 1.12)^*∗∗*^

*Note*. ^*∗*^Significant at *p* < 0.05 (crude unstandardized *β* coefficient (95% CI)). ^*∗∗*^Significant at *p*-value <0.05 (adjusted unstandardized *β* coefficient (95% CI)).

## Data Availability

The data used to support the findings of this study are available from the corresponding author upon request.

## Abbreviations

AICU: Adult intensive care unit
ARSRHs: Amhara regional state referral hospitals
ICU: Intensive care unit
NICU: Neonatal intensive care unit
PICU: Pediatric intensive care unit
RR: Respiratory rate
USA: United States of America.
